# La Aplicación Stroke Riskometer™ en la Preconsulta Hospitalaria como Campaña Educativa del Infarto Cerebral

**DOI:** 10.31083/RN44277

**Published:** 2025-06-26

**Authors:** Diego Alejandro Ortega-Moreno, Fernando Tienda-López, Egla Samantha Sánchez-Peralta, Ana Laura de León-Pérez, David Loaiza-Pérez, Fernando Chávez-Ríos, Fernando Góngora-Rivera, GECEN-Investigators

**Affiliations:** ^1^Facultad de Medicina de la Universidad Autónoma de Nuevo León, Monterrey, NL 64460, México; ^2^St. Michael’s Hospital Li Ka Shing Knowledge Institute, RADIS Lab, Toronto, ON M5B 1A6, Canada; ^3^Servicio de Medicina Interna, Hospital Universitario “José Eleuterio González”, Monterrey, NL 64460, México; ^4^Servicio de Neurología, Hospital Universitario “José Eleuterio González”, Monterrey, NL 64460, México

**Keywords:** conocimiento poblacional, factores de riesgo, ictus, México, prevención primaria, Stroke Riskometer™ App, population knowledge, risk factors, stroke, Mexico, primary prevention, Stroke Riskometer™ App

## Abstract

**Introducción:**

El ictus es altamente prevalente a nivel mundial, sin embargo, los síntomas asociados y factores de riesgo son comúnmente desconocidos en la población general. Nuestro objetivo fue describir el conocimiento de los signos tempranos de ictus y su asociación con el riesgo de infarto cerebral a 5 y 10 años según “*Stroke Riskometer™*”.

**Sujetos y métodos:**

Estudio observacional, descriptivo, transversal, incluyendo adultos de consulta externa de Neurología del Hospital Universitario “Dr. José Eleuterio González”. Se realizaron mediciones antropométricas, de signos vitales y la medición de “*Stroke Riskometer™*”, recabándose el riesgo calculado a 5 y 10 años. Se cuestionaron los signos tempranos de infarto cerebral (énfasis en acrónimo “CAMALEÓN”: CA = Cara colgada, MA = Mano pesada, LE = Lengua trabada, ON = Enciéndete y acude al hospital). Se utilizó la correlación de Spearman para medir la asociación entre riesgo y conocimiento de signos.

**Resultados:**

Se incluyeron 300 participantes, siendo 208 (69,3%) mujeres, edad promedio de 54,5 (±14,0) años. Los factores de riesgo más prevalentes de ictus fueron sedentarismo (46,3), hipertensión arterial (40,0%) y diabetes (31,0%). La mediana de riesgo poblacional a 5 años fue 3,6% (rango intercuartílico (RIC) 1,9–7,0) y a 10 años 6,3% (RIC 3,1–14,0). El 31,2% de los participantes conocía al menos 1 signo temprano de ictus. No se encontró una correlación significativa entre el conocimiento de signos tempranos y el riesgo a 5 o 10 años (r = 0,039, *p* = 0,5; r = –0,05, *p* = 0,380, respectivamente).

**Conclusiones:**

El conocimiento de los signos de ictus es bajo, manteniéndose como una meta vigente por las campañas educativas en México. Resulta necesaria una campaña nacional, masiva y permanente, ante el alto riesgo de ictus poblacional.

## Abstract (en)

**Introduction:**

Stroke is highly prevalent worldwide; however, associated symptoms and risk factors are unknown in the general population. Our aim was to describe the knowledge of early signs of stroke and its association with the risk of stroke at 5 and 10 years according to the “Stroke Riskometer™”

**Subjects and Methods:**

This was an observational, descriptive, cross-sectional study, including adults in the Neurology outpatient clinic of the University Hospital “Dr. José Eleuterio González”. Vital signs were recorded, anthropometric and the “Stroke Riskometer™” measurements were collected, and the risks at 5 and 10 yearswere calculated. Patients were questioned about the early signs of stroke (with emphasis on the acronym FAST: F = Face uneven, A = Arm hanging down, S = Speech slurred, T = Time is vital [CAMALEÓN in Spanish]). Spearman’s evaluation was used to measure the association between risk and knowledge of signs.

**Results:**

A total of 300 participants were included; 208 (69.3%) were women and the mean age was 54.5 (±14.0) years. The most prevalent risk factors for stroke were sedentary lifestyle (46.3%), high blood pressure (40.0%), and diabetes (31.0%). The population median risk at 5 years was 3.6% (interquartile range (IQR) 1.9–7.0) and at 10 years 6.3% (IQR 3.1–14.0). Of all participants, 31.2% were aware of at least one early sign of stroke. No significant correlation was found between awareness of early signs and risk at 5 or 10 years (r = 0.039, *p* = 0.5; r = –0.05, *p* = 0.380, respectively).

**Conclusions:**

Knowledge of the signs of stroke is low but remains an ongoing goal for educational campaigns in Mexico. A large-scale national and long-lasting campaign is necessary, given the high risk of stroke in the population.

## 1. Introducción

El ictus es una de las principales causas de morbilidad y mortalidad tanto México como en el mundo [1, 2]. En esta patología, el reconocimiento temprano de sus signos y síntomas asociados tiene relevancia clínica en la forma de búsqueda de atención médica oportuna, y, por lo tanto, en el desenlace clínico del paciente [3, 4, 5].

El conocimiento poblacional de los signos de alarma del ictus ha sido valorado previamente en la literatura. Una revisión sistemática que evaluó estudios previos a 2010 acerca de conocimiento poblacional de factores de riesgo de ictus, así como sus signos y síntomas tempranos de alarma, obtuvo resultados variables entre poblaciones. El conocimiento de al menos un signo clínico de ictus varió entre las poblaciones de estudio entre un 25% a 75% [6].

Más estudios similares han tenido lugar en años posteriores en distintas poblaciones [7, 8, 9, 10, 11]. Sin embargo, la población latina es una de las que más carece de estudios acerca de conocimiento del ictus [12, 13, 14]. En México existen tres estudios que evalúen conocimiento poblacional de signos tempranos y factores de riesgo, reportándose un conocimiento poblacional bajo [3, 15, 16].

El objetivo de este estudio es determinar el conocimiento de signos y síntomas de alarma tempranos de ictus en la población y su asociación con el riesgo de presentar un ictus en los próximos cinco a diez años.

## 2. Sujetos y Métodos

### 2.1 Población de Estudio

Estudio observacional, descriptivo y transversal, realizado durante las campañas de prevención organizadas en el marco del Dia Mundial del Ictus (29 de octubre). Los participantes comprendieron personas que se encontraran en la sala de espera en la consulta externa ambulatoria del Hospital Universitario “José Eleuterio González” de la Universidad Autónoma de Nuevo León, en Monterrey, México.

Se incluyeron en el estudio a todas las personas mayores de 20 años que estuvieran interesadas en conocer su riesgo de ictus. Fueron excluidas de la muestra aquellas personas que tuvieran algún grado de conocimiento médico por educación, como lo fueron estudiantes de medicina, enfermería, nutrición, biología, entre otros, así como personas que hubiesen culminado alguna de esas carreras. Asimismo, en caso de que alguna persona se realizara la medición por segunda vez, solo se tomará en cuenta la primer medición. Se eliminaron del estudio aquellas personas que se tuvieran que retirar durante la evaluación por no contar con la información completa.

### 2.2 Medición de Riesgo

Para evaluar el riego de ictus en los próximos 5 y 10 años se empleó la aplicación *Stroke Riskometer™ *(SRA, versión 3.4, AUT Ventures Limited, Auckland, Nueva Zelanda), respaldada por la *World Stroke Association* [17, 18]. La medición fue realizada por estudiantes de medicina miembros del Grupo Estudiantil Contra las Enfermedades Neurológicas (GECEN), compuesto por estudiantes de cuarto año o superiores previa capacitación por parte del Servicio de Neurología del Hospital Universitario “José Eleuterio González”. Asimismo, se recabaron las mediciones de pulso, presión arterial, ritmo y frecuencia cardiaca, oximetría digital, y medidas antropométricas como peso, talla e índice cintura-cadera.

### 2.3 Medición de Conocimiento Poblacional

Se registraron las respuestas sobre los signos y síntomas tempranos del infarto cerebral (también conocido en la población como embolia, derrame o infarto cerebral), haciendo énfasis en las respuestas que incluyan alguno de los términos del acrónimo CAMALEÓN: CA = cara colgada, MA = mano pesada, LE = lengua trabada, ON = enciéndete y acude al hospital. Una vez finalizado el cuestionamiento, se le explica de manera sencilla a las personas este acrónimo, y en caso de desconocer algún signo temprano se les entregó material impreso con información sobre estos síntomas con énfasis en la importancia de la difusión del acrónimo CAMALEÓN y la prevención de los factores de riesgo.

### 2.4 Estadística

Con propósitos ilustrativos, se subdividió la población en terciles acorde al porcentaje de riesgo según SRA (bajo riesgo, mediano riesgo y alto riesgo). Se realizó estadística descriptiva, reportando medidas de tendencia central y dispersión, empleando desviación estándar (DE) para media y rango intercuartílico (RIC) para mediana. En la estadística inferencial, se utilizó la prueba de Chi-Cuadrada para variables categóricas, U de Mann-Whitney y Kruskal-Wallis para variables numéricas, según corresponda; además la correlación de Spearman para asociación entre variables numéricas continuas. Se consideró como significativo un valor de *p* < 0,05 con un intervalo de confianza de 95%. Para el análisis, se utilizó SPSS versión 25 (Corporación IBM, Armonk, NY, USA).

## 3. Resultados

Se incluyeron un total de 300 participantes, siendo 208 (69,3%) del sexo femenino, edad promedio de 54,5 ± 14,0 años. Los factores de riesgo de ictus más prevalentes fueron sedentarismo (46,3%), hipertensión arterial (40,0%) y diabetes (31,0%). El resto de las variables demográficas se ilustran en la Tabla 1.

**Tabla 1.** **Variables demográficas y factores de riesgo**.

Variable		N = 300
Descripción antropométrica		
	Género femenino, n (%)	208 (69,3)
	Edad, media (DE)	54,5 (14,0)
	Altura en cm, media (DE)	161,5 (8,8)
	Peso, media (DE)	76,7 (16,8)
	IMC, media (DE)	29,4 (6,2)
	Índice cintura-cadera, media (DE)	0,93 (0,12)
	Presión arterial sistólica, media (DE)	123,3 (14,0)
Factores de riesgo cardiovascular		
	Consumo de alcohol, n (%)^a^	61 (20,3)
	Tabaquismo, n (%)^b^	47 (15,6)
	Hipertensión arterial, n (%)	120 (40,0)
	Sedentarismo, n (%)^c^	139 (46,3)
	Diabetes mellitus, n (%)	93 (31,0)
	Estrés en el último año, n (%)	196 (65,3)
	Enfermedad cardiaca, n (%)	54 (18,0)
	Cardiomegalia, n (%)	20 (6,6)
	Fibrilación auricular, n (%)	20 (6,6)
	Demencia o problemas cognitivos, n (%)	16 (5,3)
	Traumatismo craneoencefálico, n (%)	84 (28,0)
	Ictus, n (%)	22 (7,3)
	Antecedente familiar de primer grado ictus o infarto agudo al miocardio, n (%)	73 (24,3)
Grado máximo de estudio		
	Población sin estudios, n (%)	2 (0,7)
	Educación primaria, n (%)	49 (16,3)
	Educación secundaria, n (%)	72 (24,0)
	Educación bachillerato, n (%)	74 (24,7)
	Educación universitaria, n (%)	103 (34,3)
Ocupación actual		
	Estudiante, n (%)	11 (3,7)
	Ama de casa, n (%)	102 (34,0)
	Empleado, n (%)	122 (40,7)
	Desempleado, n (%)	15 (5,0)
	Jubilado, n (%)	50 (16,7)

^a^Consumo ocasional o mayor de alcohol, ^b^Consumo activo de al menos un cigarrillo al día, ^c^Actividad física nula en toda una semana. DE, desviación estándar; IMC, índice de masa corporal.

### 3.1 Conocimiento Poblacional de Ictus

En cuanto al conocimiento, 31,2% de la población conoce al menos un signo o síntoma del infarto cerebral. En la muestra total, los signos y síntomas presentes en el acrónimo CAMALEÓN fueron los más reconocidos: “cara colgada” (20,7%), “mano pesada” (18,0%) y “lengua trabada” (17,3%). Otro signo temprano de ictus mencionado fue cefalea (6,3%) (Tabla 2).

**Tabla 2.** **Conocimiento poblacional de los signos de alarma de ictus**.

Signos de alarma conocidos		N = 300
	Ninguno, n (%)	206 (68,7)
	1, n (%)	94 (31,2)^a^
	2, n (%)	57 (18,9)
	3, n (%)	38 (9,6)
	4 o más, n (%)	10 (3,3)
Signos más conocidos		
	Cara colgada, n (%)	62 (20,7)
	Mano pesada, n (%)	54 (18,0)
	Lengua trabada, n (%)	52 (17,3)
	Cefalea, n (%)	19 (6,3)
	Alteraciones de la coordinación, n (%)	4 (1,3)
	Alteraciones del equilibrio, n (%)	2 (0,7)
	Alteraciones sensitivas, n (%)	2 (0,7)
	Alteraciones en la conciencia, n (%)	4 (1,3)

^a^Representa el conocimiento de al menos un signo o síntoma temprano de ictus.

Existe una asociación significativa entre un mayor grado académico y el conocimiento de algún signo temprano de ictus (odds ratio (OR) = 3,409, IC 95% 1,953–5,950, *p* < 0,001), habiendo un mayor grado de conocimiento en personas que culminaron preparatoria (36,5%) o licenciatura (44,7%).

No se encontró una asociación significativa entre el conocimiento de signos tempranos de ictus y la edad por década de la vida (*p* = 0,45), antecedente personal de ictus (*p* = 0,96) o antecedente familiar de ictus (*p* = 0,36). Asimismo, no existió asociación significativa entre otro factor de riesgo y conocimiento de signos de ictus.

### 3.2 Riesgo Poblacional de Ictus

La mediana de riesgo a 5 años de la población encuestada según SRA fue de 3,6% (RIC 1,9–7,0) y a 10 años fue de 6,3% (RIC 3,1–14,0). No se encontró una correlación significativa entre un mayor conocimiento de signos tempranos de ictus y riesgo a los 5 y 10 años (r = –0,039, *p* = 0,5; r = –0,05, *p* = 0,380, respectivamente).

Existe una diferencia significativa entre el género y el riesgo de ictus a 10 años, siendo los hombres los que tienen un porcentaje mayor de riesgo en comparación a las mujeres (Mediana 8,2% RIC 5,2–15,9 vs 5,4% RIC 2,7–12,3, *p* = 0,007); a los 5 años también tienen mayor riesgo, sin embargo, no es estadísticamente significativo (Mediana 4,2% RIC 2,5–7,0 vs 3,5% RIC 1,7–6,1, *p* = 0,08).

**Fig. 1.**
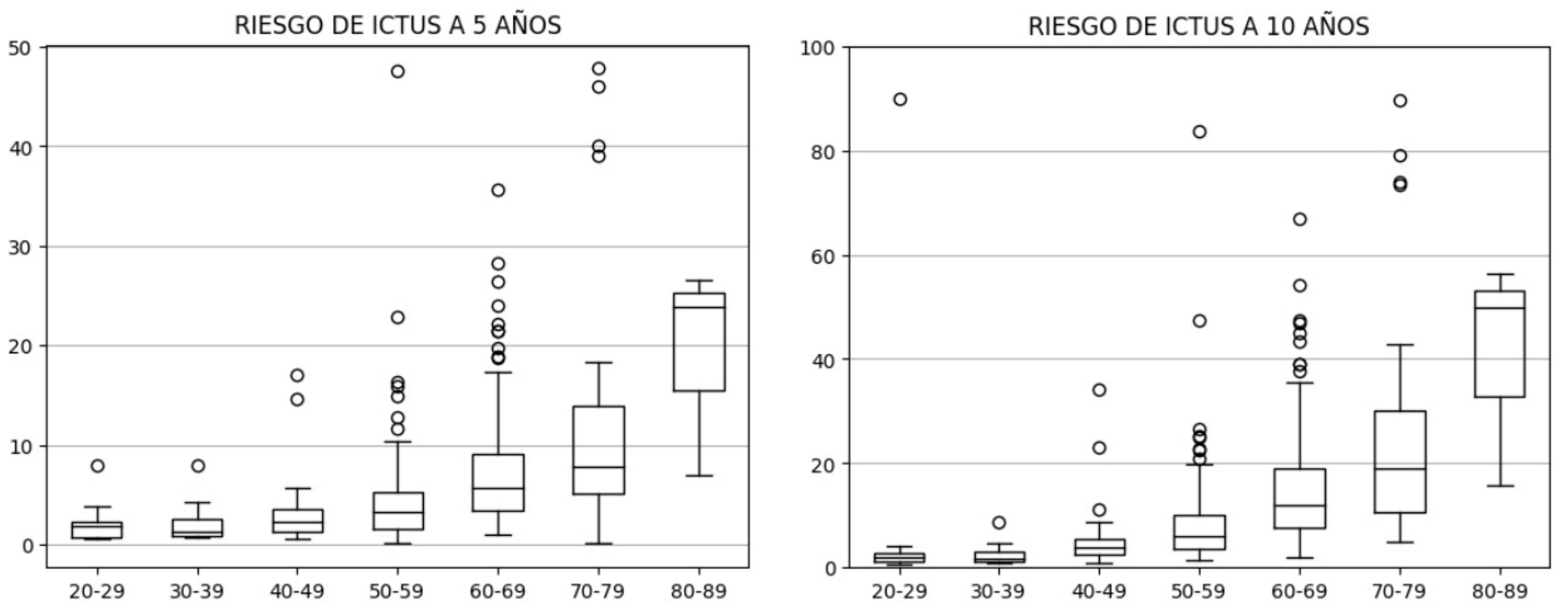
**Riesgo de ictus por década de vida**.

El riesgo a 5 y 10 años por década de la vida se ilustra en la Fig. 1. Además, se encontraron diferencias significativas entre la ocupación y el riesgo de ictus a los 5 (*p* < 0,001) y 10 años (*p* < 0,001). En el análisis *post-hoc* se aclara que es la ocupación de estudiante la que tiene menor riesgo de ictus.

No existe una asociación significativa entre el riesgo de ictus y el nivel del grado académico. Aunque existe una asociación entre mayor educación y tabaquismo positivo (OR = 2,280, IC 95% 1,131–4,596, *p* = 0,016) y ejercicio (OR = 1,636, IC 95% 1,028–2,603, *p* = 0,037), no se encontró asociación con hipertensión (*p* = 0,267), alcoholismo (*p* = 0,377) o consumo de frutas y verduras (*p* = 0,480).

### 3.3 Análisis Por Subgrupos: Bajo, Mediano y Alto Riesgo de Ictus

**Fig. 2.**
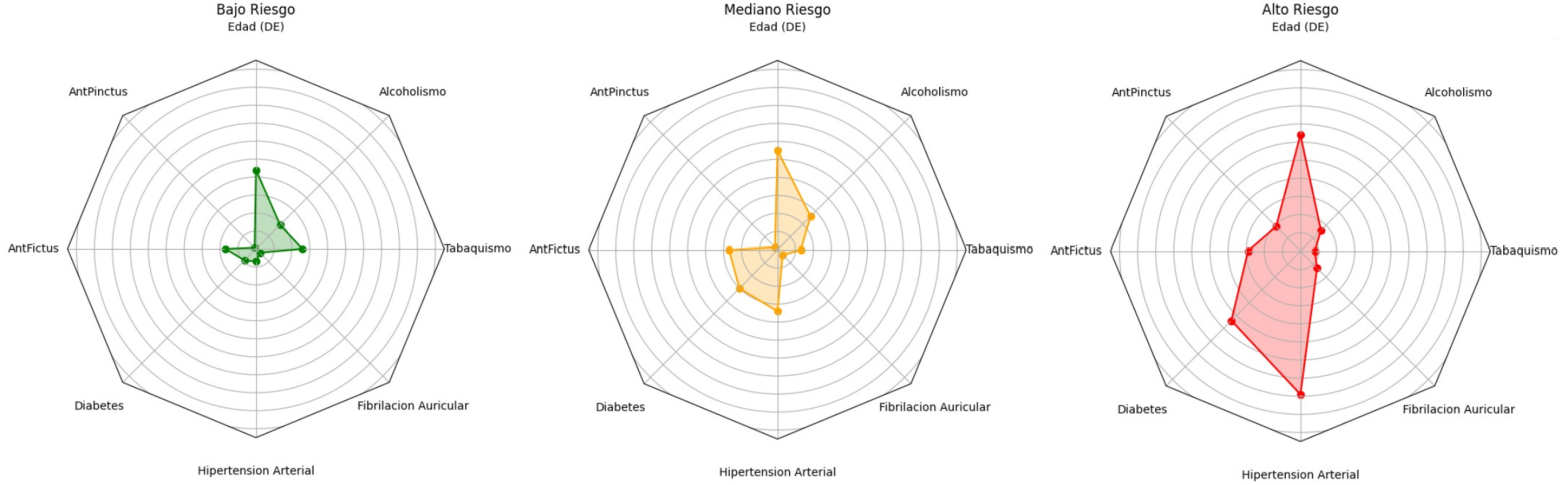
**Prevalencia de factores de riesgo en grupos de riesgo de ictus**.

La prevalencia de factores de riesgo aumenta conforme se incrementa el porcentaje de riesgo en los tres grupos (1,8 ± 1,1 vs 2,3 ± 1,3 vs 3,7 ± 1,5, *p* < 0,001, respectivamente). En el gráfico de telaraña de la Fig. 2 se ilustra la distribución y frecuencia de los principales factores de riesgo por grupo.

Aunque no existe una diferencia significativa entre los grupos según el riesgo y el conocimiento de signos tempranos de ictus (*p* = 0,705), la frecuencia de conocimientos de al menos un signo disminuye conforme aumento el riesgo (34,0% vs 32,0% vs 28,0%, *p* = 0,648).

## 4. Discusión

En nuestro análisis, existe una asociación significativa entre educación y conocimiento de al menos un signo de alarma de ictus. Sin embargo, no se encontró una correlación significativa entre el conocimiento de los signos de alarma y su riesgo a 5 y 10 años determinado por SRA. Es decir, que lamentablemente los sujetos de alto riesgo de sufrir un ictus no reconocerán mejor sus signos de alarma y que su nivel educativo es igual al grupo de menor riesgo cerebrovascular.

La SRA ha sido validada con anterioridad como un algoritmo que permite representar el riesgo de padecer un ictus en 5 y 10 años [18, 19], y por su sencillez, número de factores de riesgo a evaluar y potencial educativo, ideal para utilizar en el estudio de la población en general [20, 21]; a pesar de ello, los estudios que lo emplean son limitados. La mayoría de la literatura emplea la escala Framingham Stroke Risk Profile (FSRP) para evaluar el riesgo de ictus a 10 años [22, 23, 24], y dentro de los que han empleado SRA, Sethi *et al*. (2021) [25] evaluó el riesgo de ictus a 5 y 10 años en pacientes mayores de 50 años con diagnóstico de hipertensión arterial, encontrando una alta prevalencia de pacientes con riesgo alto a 10 años (70%) y reportando la variable tabaquismo como la que influye en mayor proporción en el riesgo de ictus.

El conocimiento poblacional de los signos y síntomas tempranos de ictus es bajo en la población mexicana (31,2% con al menos un signo), e incluso en comparación con estudios de años previos (36,7% [15], 37,6% [3] y 57,9% [16]), lo que refleja la necesidad de mantener la campaña educativa en forma permanente y oficializarla como estrategia de salud pública. Aunque hay una gran variabilidad poblacional en la referencia del signo temprano más conocido, en esta encuesta la parálisis facial fue el signo clínico más mencionado. Nuestra muestra presenta un mayor número de factores de riesgo para ictus que estudios previos, concordante con el aumento durante la década pasada de comorbilidades y sedentarismo, alcoholismo y tabaquismo en el mundo [26, 27].

En nuestro estudio, se encontró una asociación significativa entre un mayor grado académico alcanzado y un mayor conocimiento de signos tempranos de ictus. Esto es concordante con lo reportado previamente en la mayoría de los estudios que evalúan conocimiento poblacional [3, 6, 7, 8, 9, 10, 15]. A pesar de ello, como se expuso, no existió una asociación entre el grado académico y factores de riesgo modificables, excepto por el hábito de hacer ejercicio. Consideramos que es imperativo iniciar las campañas de concientización acerca de las enfermedades cardiovasculares, en especial sobre el ictus desde los niveles básicos de educación [28], para reducir la disparidad poblacional observada en este estudio. Asimismo, de gran importancia extender las campañas hacia la población rural, dado que su nivel de conocimiento, al igual que en poblaciones urbanas, es bajo [29, 30].

La literatura previa asoció el antecedente personal o familiar de ictus con un mayor conocimiento de signos de alerta, lo cual puede explicarse por el aprendizaje derivado de la experiencia al interior de la familia [3, 9, 10]. La población encuestada tuvo menos exposición a esta enfermedad, pudiendo ser un factor que posiblemente influyó en el menor conocimiento de los signos de alerta del ictus.

A través de la iniciativa representada en el presente estudio se buscó promover en la población general el conocimiento del ictus, describiendo y explicando al participante sus factores de riesgo asociados, los signos principales del ictus isquémico, el riesgo del participante de desarrollar un ictus a 5 y 10 años según SRA, así como recomendaciones generales para la promoción individual de la salud. No existe duda en la importancia de las estrategias enfocadas en la educación poblacional para la reducción de la prevalencia de ictus isquémico, así como para la búsqueda oportuna de atención médica en el caso de desarrollarlo. Estas estrategias, en concordancia con los objetivos del presente estudio, buscan la concientización poblacional acerca del ictus. Sin embargo, debemos destacar que, a pesar de los continuos esfuerzos a nivel regional y mundial para la promoción de los factores de riesgo y signos tempranos del ictus en la población, estas estrategias aisladas son insuficientes para la prevención del ictus, haciendo necesarias campañas permanentes a través de medios de comunicación masiva y redes sociales para poder generar un mayor impacto global. Estas estrategias deberán de ser respaldadas y promovidas por organizaciones enfocadas en la salud, así como por el gobierno. Resulta interesante buscar la evaluación de las estrategias implementadas en las distintas poblaciones, describiendo el conocimiento de las características previamente descritas del ictus, y el impacto en el conocimiento poblacional que la implementación de las estrategias produciría.

## 5. Limitaciones

Resulta importante destacar que la metodología utilizada para la recolección de los datos recabados fue a través de la evaluación directa persona-persona, lo cual limita el número de encuestas. Para incrementar el uso de la SRA, se recomendó que cada persona participante en la encuesta la instalara en sus teléfonos portátiles y nos ayudara a difundirla. Consideramos que en nuestro país aún faltan esfuerzos para incrementar el uso de estas estrategias digitales, que han sido útiles en otros países.

La muestra poblacional es pequeña, pero es representativa del medio hospitalario público en México, y logró incluir una población diversa desde el punto de vista demográfico y educativo.

## 6. Conclusiones

En base a los hallazgos representados, se evidenció que, a pesar de las continuas estrategias de educación locales y regionales, incluso en la población de mayor riesgo de sufrir un ictus, se requiere incrementar el conocimiento de la enfermedad con énfasis en los signos de alarma para mejorar su atención oportuna.

## Data Availability

Los conjuntos de datos utilizados y/o analizados durante el presente estudio están disponibles a pedido razonable del autor correspondiente.

## Divulgación

El presente trabajo ha sido previamente presentado en formato póster en el congreso de la Asociación Mexicana de Enfermedad Vascular Cerebral (AMEVASC) y como presentación oral y resumen en el International Journal of Medical Students.

## Contribuciones de los Autores

Conceptualización: DAOM, FTL, ESSP, DOR, ENJ; Curación de datos: DAOM, FTL, ESSP, ENJ; Análisis Formal: FTL, FGR, ENJ; Investigación: FTL, ESSP, ALLP, DLP, FCR, SSR, ARS; Metodología: DAOM, ESSP, ALLP, DLP, FCR, FGR, SSR, DOR, ARS, ENJ; Administración de Proyecto: DAOM, FTL, ALLP, DLP, FCR, FGR, DOR, ARS; Supervisión: FGR, SSR, DOR, ARS; Validación: FTL, SSR; Redacción – manuscrito original: DAOM, FTL, ALLP, DLP, FCR; Redacción – edición manuscrito final: FTL, FGR. Todos los autores contribuyeron a los cambios editoriales en el manuscrito. Todos los autores leyeron y aprobaron el manuscrito final. Todos los autores han participado lo suficiente en el trabajo y han acordado ser responsables de todos los aspectos del mismo.

## Aprobación Ética y Consentimiento Informado

Todos los participantes en el proyecto dieron su consentimiento verbal para su inclusión en el estudio previo a su medición de riesgo. El consentimiento informado oral ha sido aprobado por el comité de ética. El estudio fue llevado a cabo de acuerdo con la Declaración de Helsinki, y el protocolo fue aprobado por el Comité de Ética en Investigación del Hospital Universitario “Dr. José Eleuterio González”, clave de registro MI25-00001.

## Agradecimientos

Jorge Guadalupe Rivera-Trujillo por apoyo en la elaboración de las imágenes.

## Financiación

Esta investigación no recibió financiación externa.

## Apéndice

**Apéndice 1 – GECEN-Investigators**

Samantha Salinas-Rodríguez^1^, Diego Oyervides-Recio^1^, Arely Rodríguez-Salazar^1^, Emanuel Navarrete-Juárez^1^
